# Different Effects of Resveratrol on Dose-Related Doxorubicin-Induced Heart and Liver Toxicity

**DOI:** 10.1155/2012/606183

**Published:** 2012-11-26

**Authors:** Jaroslaw Dudka, Renata Gieroba, Agnieszka Korga, Franciszek Burdan, Wlodzimierz Matysiak, Barbara Jodlowska-Jedrych, Slawomir Mandziuk, Elzbieta Korobowicz, Marek Murias

**Affiliations:** ^1^Medical Biology Unit, Medical University of Lublin, 20-059 Lublin, Poland; ^2^Human Anatomy Department, Medical University of Lublin, 20-059 Lublin, Poland; ^3^Histology and Embryology Department, Medical University of Lublin, 20-059 Lublin, Poland; ^4^Department of Pulmonolgy, Oncology and Alergology, Medical University of Lublin, 20-059 Lublin, Poland; ^5^Clinical Pathomorphology Department, Medical University of Lublin, 20-059 Lublin, Poland; ^6^Department of Toxicology, Poznan University of Medical Sciences, 60-780 Poznan, Poland

## Abstract

The aim of the study was to evaluate the effect of resveratrol in doxorubicin-induced cardiac and hepatic toxicity. Doxorubicin was administered once a week throughout the period of 7 weeks with 1.0 or 2.0 mg/kg body weight or concomitantly with resveratrol (20 mg/kg of feed). Heart and liver toxicity was histologically and biochemically evaluated. Resveratrol protected from the heart lipid peroxidation caused by 1 mg doxorubicin and it sharply diminished superoxide dismutase activity. An insignificant effect of resveratrol on the lipid peroxidation level and the superoxide dismutase activity was observed in the hearts of rats administered a higher dose of doxorubicin. However, resveratrol attenuate necrosis and other cardiac histopathological changes were induced by a high dose of doxorubicin. Interestingly, it slightly intensified adverse cardiac histological changes in rats receiving a lower dose of doxorubicin. Resveratrol did not have any protective effect on the hepatic oxidative stress, while exerting a mild beneficial effect on the morphological changes caused by doxorubicin. All in all, this study has shown different effects of resveratrol on dose-related doxorubicin-induced heart and liver toxicity. Resveratrol may modulate the hepatic and cardiac effect of doxorubicin, depending on the drug dose.

## 1. Introduction

Drug-drug and drug-food interactions are specially important in the anticancer therapy because of the very narrow therapeutic index of these chemotherapeutics. Moreover, many patients suffering from cancer in the terminal state decide to take unconventional agent, very frequently beyond of a doctor knowledge.

Doxorubicin (DOX) is a very efficient antitumor drug, but its administration is limited by a dose-dependent, irreversible, and progressive cardiomyopathy, which may become evident years after completion of the therapy [1–5]. The pathomechanism of DOX-related late cardiotoxicity is multifactorial [6, 7], but the prevalent hypothesis ascribes the dominant role to oxidative stress linked to redox-cycling of the drug [8, 9]. The DOX redox-cycling is started from one-electron reduction with the formation of DOX radical (DOX*) [10]. Many NADPH and NADH-dependent enzymes catalyze that reaction, for example, NADPH cytochrome P450 reductase [9], NOS [11–13], NADPH oxidase [14, 15], and catalase [16]. Subsequently, DOX* are reoxygenated to the nonradical parent compound while at the same time the superoxide anion radical is formed (O_2_^−∗^). This cycle of reactions may repeat many times leading to overproduction of O_2_^−∗^, which is the source of hydrogen peroxide and much more toxic hydroxyl radical [6, 17]. These reactive oxygen species (ROS) are responsible for oxidative stress. The above-mentioned enzymes involved in DOX* production are abundant in hepatocytes [18], suggesting that liver may be specially involved in DOX* generation. Although no such intense DOX synthase takes place in the heart, relatively low antioxidant defense of cardiomyocytes makes the heart a target for DOX toxicity [19]. The studies of the last decade have been focussed towards the understanding of the latent period of DOX-mediated cardiomyopathy caused by oxidative stress. Redox cycling transformation occurs in cytoplasm, endoplasmatic reticulum, and specially in mitochondria [20]. Oxidative changes in mitochondrial DNA are responsible for disturbances in mitochondrial protein synthesis. For that reason, the product of the electron transfer chain is not only four electron reduced oxygen (H_2_O) but also ROS—one, two, and three electron products—O_2_^−∗^, H_2_O_2_, and HO*, respectively [21]. On the other hand, the oxidation of mtDNA by ROS appears again. In the beginning, the symptoms during that latent period are not clinically overt [22, 23]. Insufficiency of mitochondria function becomes more visible with the pass of time. For that reason, participation of glycolysis pathway in ATP synthesis arises. Eventually, after months or years, the death of cardiomyocytes [24, 25] can consequently lead to heart failure [26, 27]. 

Resveratrol (3,5,4′-trihydroxystilbene) is a polyphenol, presents in some plants growing in Mediterranean countries (grapes), Minor Asia (hellebore), and Japan (knotweed), is easy achievable as a diet supplement. Resveratrol has been studied in many clinical trials [28, 29] and has shown anticancer [30–32], cardioprotective [33–35], and antioxidant activity [36–39]. Previous studies demonstrated that resveratrol causes resistance to oxidant injury in rats' neonatal cells (H9c2) [40], and it prevents from cardiomyocytes' cytotoxicity of DOX through mitochondrial stabilization [41].

Thus, resveratrol having antioxidative and anticancer activity can be additionally taken as a supplement by the doxorubicin-treated patient. The aim of this study was to verify the hypothesis according to which resveratrol protects against oxidative injury and pathomorphological changes caused by DOX.

## 2. Materials and Methods

### 2.1. Animals and Treatment

The experimental protocol was approved by the Local Bioethical Council of the Medical University in Lublin. The study was conducted on sexually mature male albino rats of Wistar CRL:(WI)WUBR strain, obtained from a commercial breeder (Warszawa-Rembertow, Poland). Animals with the initial body weight of 160–195 g were maintained in stable conditions at 22°C with a 12 h light/dark cycle and given standardized granulated fodder LSM (AGROPOL, Poland). The rats were exposed to doxorubicin (DOX; Ebewe, Austria) and resveratrol (RV, Sigma-Aldrich, USA). The animals were randomly divided into control (*n* = 8) and 4 study groups (*n* = 6): 1DOX and 2DOX where doxorubicin was injected intraperitoneally alone in a dose of 1.0 or 2.0 mg/kg body weight, respectively. In groups 1DOX+RV and 2DOX+RV, doxorubicin was administered according to the same scheme concomitantly with resveratrol given with the fodder LSM prepared by AGROPOL in the concentration 20 mg/kg of fodder (20 ppm). In the control group, a 0.9% NaCl was intraperitoneally injected. Doxorubicin was dissolved with saline 1 : 2 v/v and injected once a week for seven weeks in all study groups. Dietary resveratrol supplementation was started one week before the administration of doxorubicin and followed throughout the study. Since in most of previous studies [42, 43] resveratrol in the selected dose (20 mg/kg) did not show any significant effect on hepatic and cardiac morphology and function, secondary to the suggestion of the local Bioethical Committee the group exposed exclusively to resveratrol was not designated. 96 hours after administration of the last dose of doxorubicin, the animals were anesthetized by the intraperitoneal administration of 1 mg/kg pentobarbital, and the blood was collected from the left cardiac ventricle. Immediately after decapitation, the heart and liver samples were collected during autopsy. The organs were washed with saline and then the heart was sectioned along the interventricular and coronal grove. The wall of the left ventricle and the liver samples were placed in liquid nitrogen and stored at −75°C until the time of biochemical analysis. The right ventricular wall and the liver samples were fixed in buffered 10% formalin and routinely histologically processed to paraffin blocks.

### 2.2. Serum and Plasma Parameters

The concentrations of both rat heart fatty acid binding protein (H-FABP, Life Diagnostic, USA) and rat brain natriuretic peptide (BNP, Phoenix Pharmaceuticals, USA) were determined, respectively, in rat serum and plasma, using ELISA commercial kits. In both cases, two types of antibodies were used in the evaluation: antibodies covering microtiter plate and the secondary antibodies bound to horseradish peroxidase. The products of the catalytic reactions were spectrofotometrically detected at 450 nm.

Serum activity of aspartate aminotransferase (AST), alanine aminotransferase (ALT), kreatine kinase CK, lactated dehydrogenase (LDH) and alkaline phosphatase (ALP) was determined using standard kits of CORMAY (Poland).

### 2.3. Cardiac and Hepatic Oxidative Markers

All measurements were conducted on homogenates obtained from ~20 mg of frozen cardiac or hepatic samples using the extraction buffer provided by the manufacturer of each commercial kit. The evaluation of lipid peroxidation in cardiac and hepatic homogenates was based on malondialdehyde and 4-hydroxyalkenals concentration (MDA+4HAE) using the commercial kit (Bioxytech LPO-586; OxisResearch, USA). The concept of the method is based on the reaction between MDA and 4HAE with N-methyl-2-phenylindol. After mixing N-methyl-2-phenylindole and methanol with the supernatant acquired from the homogenization, methanesulfonic acid was added and all reagents were placed at the temperature of 45°C for 60 minutes. Next, the solution was centrifuged and the supernatant containing the product was transferred to the plastic plate used in the spectrophotometric reader PowerWave XS (BioTek, USA) at 586 nm. Subsequently, the procedure was conducted according to the manufacturer's description and the concentration of MDA+4HNE in the tested samples was calculated from the formula of the calibration curve *y* = 0.0896*x* − 0.008. The obtained date was calculated taking into account the recommendation described in the procedure. The obtained results were expressed in nmol/g cardiac or hepatic sample.

 Glutathione determination was conducted using a commercial kit Bioxytech GSH/GSSG-412 (OxisResearch, USA). The frozen cardiac or hepatic samples (~20 mg) were homogenized in the extraction buffer provided by the manufacturer. Total glutathione (GSH_T_: GSH (reduced glutathione) + GSSG (oxidized glutathione)) was determined in the enzymatic reaction, where Ellman's reagent (5,5′-dithiobis-2-nitrobenzoic acid) reacts with GSH forming a color product with the maximum of absorbance at 412 nm. The concentrations of GSH, GSSG, and GSH/GSSG ratio were assessed after measuring the speed of the reaction and establishing the calibrations curves. The concentrations of GSH and GSSG were determined based on the calibration curve described by the formulae: *y* = 0.1447*x* + 0.0004 and *y* = 0.1475*x*, respectively. The obtained data was used to calculate the GSH/GSSG ratio.

The activity of superoxide dismutase activity (SOD) was determined colorimetrically using Bioxytech SOD-525 kit (OxisResearch, USA). The method is based on SOD-mediated increase in the rate of autoxidation of 5,6,6 a,11b-tetrahydro-3,9,10-trihydroxybenzo[c]fluorine in alkaline solution to yield chromophore with the maximum of absorbance at 525 nm. 

The activity was measured by kinetic spectrophotometric plate reader using a PowerWave XS (BioTek, USA). The mean value in the proper control groups (for cardiac or liver samples) was assumed to be 100%. 

### 2.4. Preparation of Slides for Histological Evaluation

4 *μ*m histological slides obtained from paraffin blocks were routinely processed and stained with hematoxylin and eosin (H&E). To visualize cardiomyocyte necrosis, Selye's method was also used. Liver slides were also stained with van Gieson, paS (periodic acid-Schiff), and d-paS (diastase + paS).

### 2.5. Statistical Analysis

The obtained data was expressed as mean ± SD and analyzed by STATISTICA 5.0 software. Continuous data were compared among the experimental groups using the Kolmogorov-Smirnov test. The statistical significance of differences between control and study groups was evaluated by Student's *t*-test or Mann-Whitney *U* test. Group-to-group comparisons were made by one-way ANOVA. A value of *P* < 0.05 was considered as statistically significant.

## 3. Results

Lack of animal mortality was found during the study. Food and water consumption, as well as body weight gain, were insignificant between the xenobiotic-exposed and control groups. A significant increase of FABP and BNP levels was observed in rats exposed to the higher dose of doxorubicin (Table 1). Moreover, resveratrol significantly reduced the concentration of both parameters in animals receiving a higher dose of doxorubicin to the levels below of control.

Aspartate aminotransferase (AST) activity was significantly decreased in the group concomitantly exposed to a higher dose of doxorubicin and resveratrol. On the other hand, alanine aminotransferase (ALT) activity was increased in animals exposed to both resveratrol and a low dose of doxorubicin but decreased among the rats treated with resveratrol and a high dose of the drug. A statistically significant decrease of lactate dehydrogenase (LDH) activity was noted in the groups exposed to a high dose of doxorubicin with or without resveratrol. In the case of creatine kinase (CK) activity, an significant change was also revealed in the group treated with resveratrol and a higher dose of the drug. A significant decrease of the ALP activity was found in all the xenobiotic-exposed groups. However, no significant changes in the activity of all the above-mentioned enzymes were noticed between the groups the DOX+RV versus DOX group.

**Figure 1:**
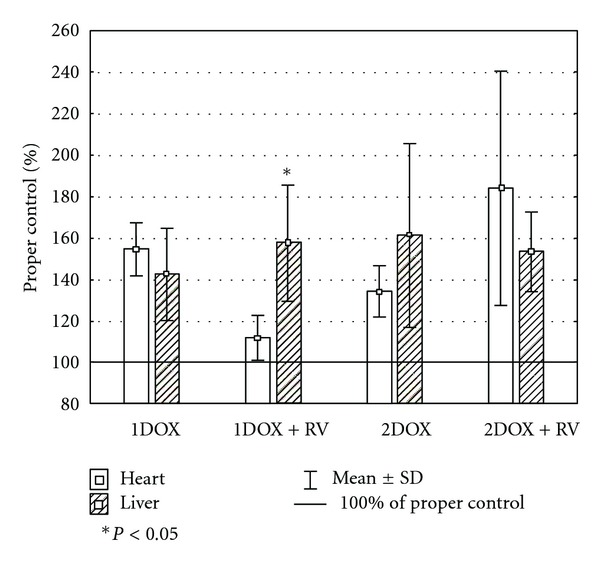
The relative differences of MDA+4HNE between heart and liver.

**Figure 2:**
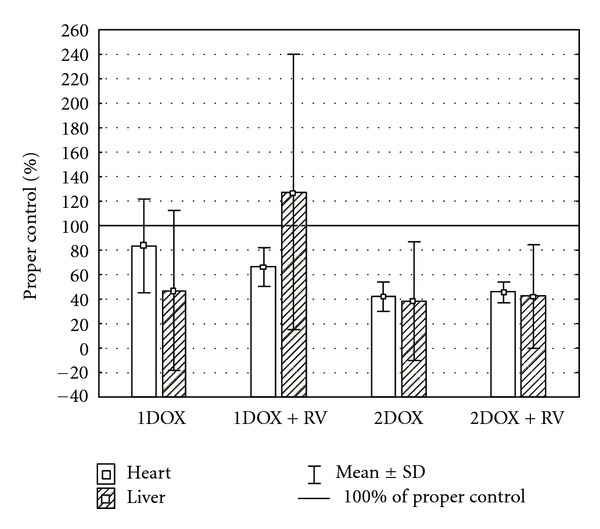
The relative differences of GSH/GSSG between heart and liver.

**Figure 3:**
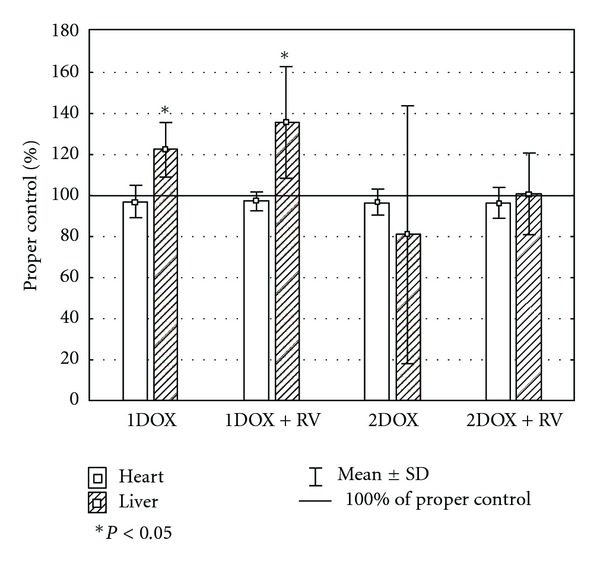
The relative differences of GSH_T_ between heart and liver.

**Figure 4:**
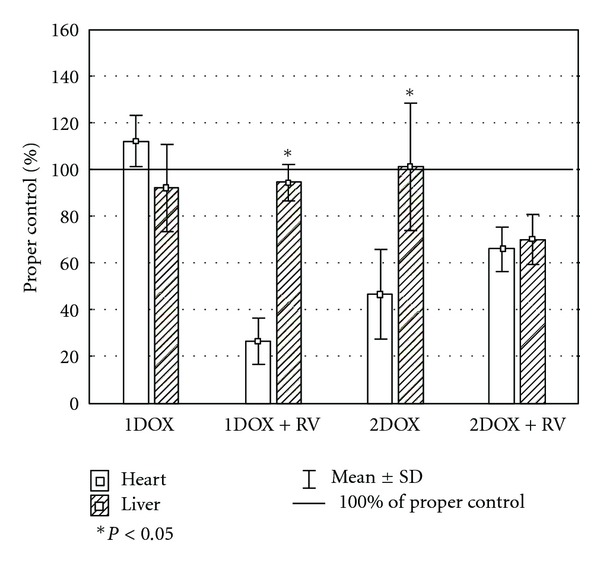
The relative differences of SOD between heart and liver.

Unlike the insignificant changes of GSH_T_ in cardiac homogenates, all other parameters of oxidative stress were highly affected by the tested substances (Table 2). Significant changes of MDA+4HAE, GSH/GSSG ration, and SOD were found in groups exposed to a high dose of doxorubicin with or without resveratrol. An increase of MDA+4HAE was also noted among the animals exposed exclusively to a low dose of doxorubicin. A decrease of SOD activity was found in the group cotreated with resveratrol and a low dose of the drug as well. The value was also significantly lower when compared to the group exposed only to a low dose of doxorubicin. A similar effect among the groups 1DOX+RV and 1DOX were found in case of MDA+4HAE. Insignificant differences in hepatic GSH_T_, and SOD level were found in all studied groups comparing to the control. However, the GSH/GSSG ratio was significantly lower in both groups receiving a higher dose of doxorubicin versus control (Table 3), while MDA+4HNE was significantly higher in all tested groups comparing to the control. No significant changes in liver-determined oxidative markers between the DOX+RV versus DOX group were found. Several complex changes in relative values of oxidative stress markers between heart and liver were observed only in the group of RV+1DOX (Figures 1, 2, 3, and 4). These rats had significantly higher relative changes in liver MDA+4HNE, GSH_T_ and SOD level than in the heart. A similar result referring to GSH_T_ was found in 1DOX and referring to the SOD in 2DOX. There were no significant changes between heart and liver in GSH/GSSG relative values in all studied groups.

**Figure 5:**
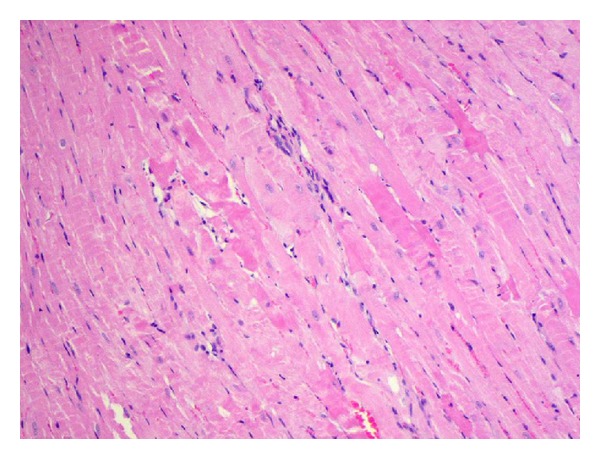
Interstitial edema and inflammatory infiltration between irregualar wavy-directed cardiomiocytes (H and E; objective mag. ×20; group 2DOX).

**Figure 6:**
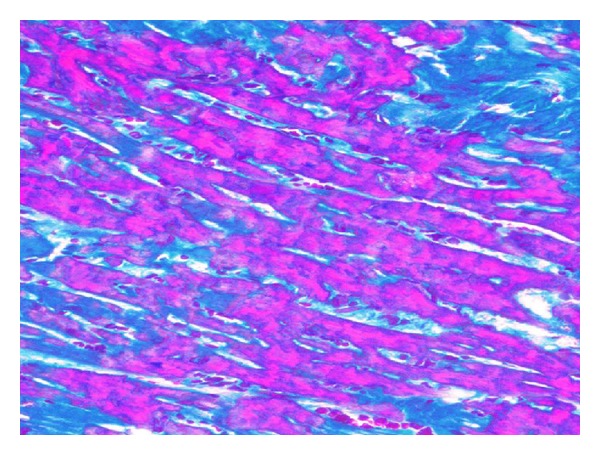
Necrosis of cardiomiocytes (Selly's staining; objective mag. ×40; group 2DOX).

**Figure 7:**
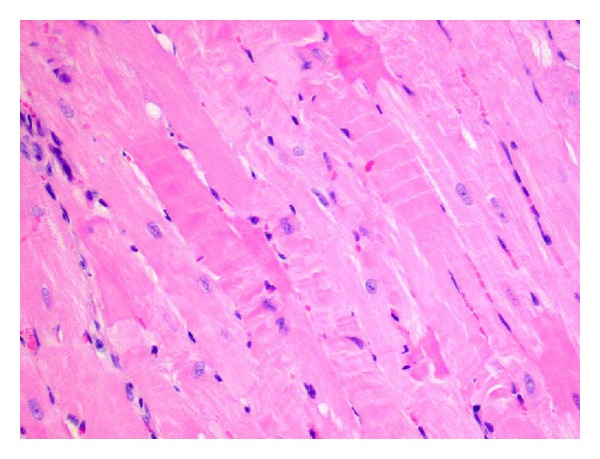
Intestitial edema eosynophylic cytoplasm and inflammatory infiltration between irregular wavy-directed cardiomiocytes (H and E; objective mag. ×40; group 2DOX+RV).

Histological cardiac abnormalities in the untreated control group were limited mostly to single cases of irregular, wavy direction of cardiomyocytes (Table 4). Occasionally, a concomitant parenchymatous degeneration and interstitial edema were also revealed. The highest occurrence of pathological changes was found in groups exposed exclusively to doxorubicin (Figure 5). The effect was dose-dependent since massive necrosis was found only in 4 of 6 animals exposed to the high dose of the drug (Figure 6). In the remaining two animals and in 4 out of 6 ones from the group treated with a low dose of doxorubicin, the necrosis was limited to single, spread cardiomyocytes. In all cases an inflammatory infiltration was located around necrotic loci. Dose dependence was also confirmed in case of the eosinophilic degeneration and interstitial edema. A lower incidence but similar histological changes were observed in groups concomitantly treated with doxorubicin and resveratrol (Figure 7). Insignificantly higher occurrence of the eosinophilic degeneration was found in the group cotreated with resveratrol and a low-dose doxorubicin, when compared with rats exposed only to the low dose of the drug, while a lower incidence of such abnormality was revealed in animals exposed to resveratrol and a high dose of doxorubicin. However, the incidence of necrosis and inflammatory infiltration was lower than that in the groups exposed only to doxorubicin.

**Figure 8:**
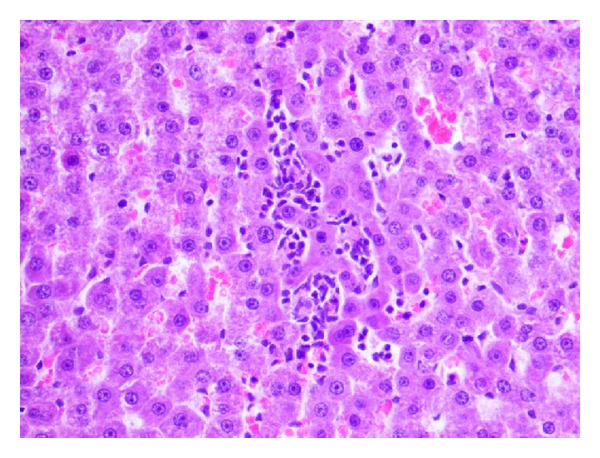
Eosinophilic degeneration and local inflammatory infiltration in a central zone of the hepatic lobule. Pycnotic nuclei in selected hepatocytes (H and E; objective mag. ×40; group 2DOX).

**Figure 9:**
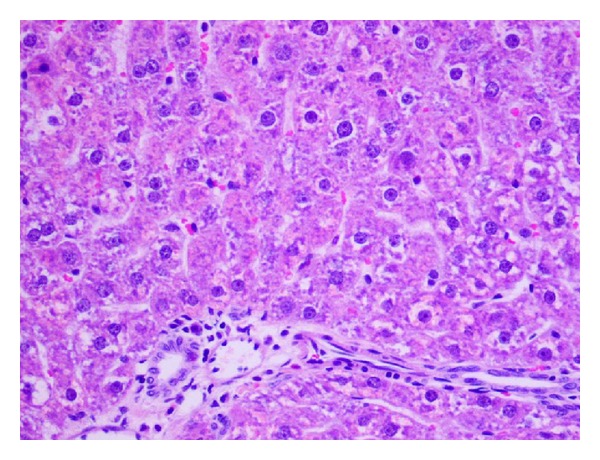
Parenchymatous and vacuolar degeneration in a peripheral zone of the hepatic lobule with a lesser foci of mononuclear inflammatory cells (H and E; objective mag. ×40; group 2DOX).

**Figure 10:**
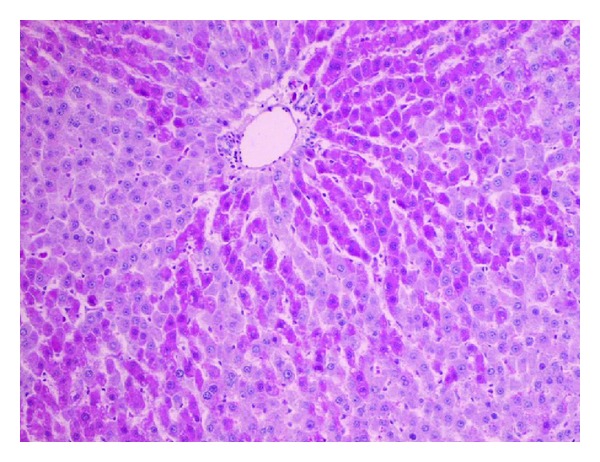
Irregular positive cytoplasm p.a.S reaction in the middle and central zone of the hepatic lobule (p.a.S.; objective mag. ×20; group 2DOX).

**Figure 11:**
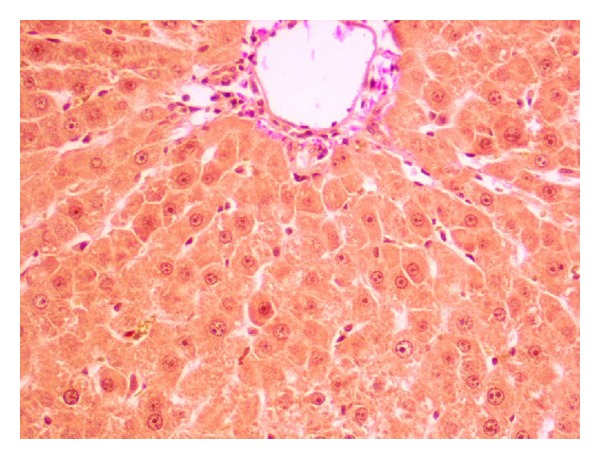
Single fibers of the connective tissue around the central vein. Pycnotyc nuclei in selected hepatocytes in the central zone of the hepatic lobule (van Gieson's; objective mag. ×40; group 1DOX-RV).

Hepatic changes were occasionally observed in the control group (Table 5). The parenchymatous (Figure 8) and eosinophilic hepatic degeneration (Figure 9) was the most commonly observed among all the xenobiotic-exposed groups. Usually, they were limited to centri- and midlobular zones. Activation of cells lining to sinus zone was also observed in such cases. Many disseminated eosinophilic hepatocytes presented an intensive cytoplasmic paS reaction (Figure 10). Moreover, d-paS staining performed in the paS-positive cases and in organs with the eosinophilic degeneration showed an extracytoplasmic, intrahepatic capacity, which corresponds to the glycogen accumulation. However, some hepatocytes with the eosinophilic degeneration did not stain in the paS method before and after diastase digestion. The highest but similar occurrence of the parenchymatous and eosinophilic hepatic degeneration was found in two groups treated exclusively with doxorubicin. However, the vacuolar degeneration and pycnotic nuclei of hepatocytes were observed only in rats treated with a high dose of the drug and in groups exposed to both examined xenobiotics. The occurrence of hepatic changes was insignificantly lower among the animals co-treated with doxorubicin and resveratrol. Unlike that in the case of the hearts, lack of necrosis was found in the examined livers but occasionally some local accumulation of mononuclear inflammatory cells, mainly in mid- and centrilobular zone, was seen. In two animals exposed to the high dose of doxorubicin and resveratrol, an increased amount of the fibrous connective tissue around the central vein was found (Figure 11). There were no features of cholestasis or fibrosis in van Gieson's staining.

## 4. Discussion

As it was expected, DOX in the two tested doses induced oxidative stress in rat heart and liver. The protective effect of resveratrol against lipid peroxidation was only found in the heart of rats exposed to a lower dose of DOX. It is worth to stress that at the same time resveratrol slightly intensified adverse cardiac histological changes in rats receiving a lower dose of DOX, but it also attenuated necrosis and other histopathological changes in the heart induced by a higher dose of the drug. Moreover, resveratrol had no protective influence on the liver oxidative stress and while having a mild beneficial effect on the organ morphological changes caused by doxorubicin.

In this study, DOX was administered 7 times, every next week in the doses 1.0 or 2.0 mg/kg, which according to other studies represents no observed general toxic effect and general toxic effects without mortality, respectively [23]. However, a similar schedule using even 0.8 mg doxorubicin/kg results in adverse cardiac effects on the histological, ultrastructural and biochemical level [44]. According to the current hypotheses referring to prolonged DOX-dependent cardiotoxicity, oxidative damage caused directly by DOX leads to mitochondrial DNA (mtDNA) damage, which in turn is responsible for mitochondrial dysfunction [23]. No changes in mitochondrial electron transfer result in oversynthesis reactive oxygen species [45]. For that reason we assessed the oxidative stress marker (MDA+4HNE) 96 hours after the last seventh dose of the drug, since the intramyocardial half-life of DOX appears to be only a few hours [46].

In this study resveratrol was taken by the rats in the fodder (20 ppm). Assuming that the average daily amount of fodder taken by rats weighing 100 g is 10 g, the dose of resveratrol is 2.0 mg/kg. It is a very low dose because NOAEL (no observed adverse effect level) for resveratrol estimated in a 28-day test, when rats were administered every day intragastrically, equals 300 mg/kg [43]. Clinically, a well-tolerated dose of resveratrol is up to 5 g/day (approximately 70 mg) [28]. In the studies with resveratrol the usually used doses are within the range of 3 mg/kg−120 mg/kg body weight or 5 g/kg of fodder [47–50].

To evaluate DOX cardiac and hepatic toxicity and to assess the effect of resveratrol on those changes, the markers of oxidative stress, histopathological features, and especially necrosis and blood biochemical parameters, were analyzed. Among the oxidative stress parameters the cardiac and hepatic lipid peroxidation products (MDA+4HNE), GSH/GSSG ratio, total glutathione GSH_T_ and activity of superoxide dismutase were determined. Resveratrol was found to have a significant protective role in the heart, since lipid peroxidation induced by a lower dose of doxorubicin was diminished in the group DOX+1RV to the level seen in the control. Similarly, residual activity of SOD—the key enzyme neutralizing O_2_^−∗^, generated by doxorubicin was lower in cardiac homogenates in group 1DOX+RV than that in 1DOX group. The rational explanation of the SOD phenomena is difficult, especially in the light of a lack of such changes comparing 2DOX+RV with 2DOX. However, the aforementioned protective role of resveratrol referring to lipid peroxidation may be attributed to the ROS scavenging activity of resveratrol which was confirmed by Murias et al. [51] and Leonard et al. [52]. No sign of the protective effect of resveratrol against the cardiac lipid peroxidation of rats administered with a higher dose of doxorubicin (2DOX versus 2DOX+RV) may result from ROS overproduction, which cannot be composed by resveratrol. According to the study by Kitada et al. [53], resveratrol can improve the antioxidative potential via reduction of tyrosine-nitrated modification of SOD, but the applied dose was 1500-fold higher than that in this study. The absence of the effect of phytophenol on the oxidative stress in the liver induced by doxorubicin may have a similar background. There is much higher activity of enzymes taking part in one electron reduction of DOX in the liver [54]. Thus, abundance of ROS caused oxidative stress cannot be scavenged by resveratrol. Moreover, resveratrol undergoes intensive metabolism by sulfotransferases and glucuronosyltransferases highly expressed in the liver [55].

Different effects of resveratrol on cardiac histology were observed. However, it generally depended on the doxorubicin dose. Resveratrol strengthened the morphological adverse changes in the heart of rats given a lower dose of DOX and attenuated the pathological features when the dose of the chemotherapeutic drug was higher. The reasonable explanation of that is that resveratrol in some redox conditions may act as an antioxidative but in other conditions as a prooxidative factor. However, comparing the DOX versus DOX+RV groups, it may be found that changes in the morphological features of the heart are not accompanied by oxidative stress, thus, oxidative stress of the rats administered with DOX and resveratrol plays an important but not a crucial role in morphological changes. It was clearly seen in the groups 2DOX+RV, when MDA+4HNE concentration was the highest among all the tested groups, but necrosis in the heart estimated histopathologically and biochemically (FABP) was significantly reduced. Moreover, the normalizing effect of resveratrol was also found referring to the contractility function. A higher dose of doxorubicin significantly elevated blood BNP level, but resveratrol given together with 2DOX significantly reduced BNP level. On the basis of the current knowledge, it is difficult even to speculate about the reason for that. On the other hand, there were no features of necrosis in the liver, but resveratrol showed a protective effect against other observed morphological changes in that organ.

Furthermore, there were no significant differences in serum activity of LDH, CK, AST, ALT, and ALP between, DOX and DOX+RV group. However, in the group 1DOX+RV, the level of CK, and in the group 2DOX + RV the levels of AST and ALT were lower than control in contrast with DOX groups, where there were no significant differences comparing to the control.

The range of changes in red-ox parameters may differ between the heart and the liver. In the group of 1DOX+RV, significantly higher relative changes in liver MDA+4HNE, GSH_T_ and SOD level comparing to the heart were found. However, there were no such complex changes between the heart and the liver in the other groups. The changes in MDA+4HNE level indicate that oxidative stress is stronger in the liver than in the heart and a higher level of GSH_T_ and SOD may be interpreted as an adaptation feedback on oxidative changes. In conditions of the conducted study, the effect of oxidative/antioxidative changes in the organs is dependent mainly on two mechanisms the first being the efficiency of xenobiotic-dependent ROS overproduction and the second, the potential of antioxidative organ defence. The liver, because of the xenobiotic metabolism, may produce an important amount of ROS but at the same time the activity of antioxidative defence is a few times higher than, for example, that in the heart [56–58]. Probably, the difference between the heart and the liver in the group of 1DOX+ RV results predominantly from the metabolism of doxorubicin and resveratrol by the liver. Both are metabolised by the monooxygenase system (cytochromes P450) which produces ROS [18, 59]. Interestingly, there were no morphological feature of necrosis in the liver contrary to the heart, where necrosis was observed in all the studied groups.

In the light of this study, the questions arise why a higher hepatic oxidative stress comparing to the heart did not cause necrosis and why despite a higher oxidative stress in the liver, the target of long-term toxicity is the heart and not the liver. Moreover, this comparison may indicate that oxidative stress is not a major necrosis predictor. The compartmentalization of the subcellular oxidative stress seems to be reasonable to explain these questions. Although, superoxide radical may freely diffuse through intracellular membranes, subcellular compartmentalization of glutathione may significantly modulate the harmful activity of ROS in subcellular compartments [60]. Therefore, probably more important is not the sum of oxidative stress in all cell organelles, as was measured in this study, but the level of mitochondrial oxidative stress. According to this assumption, a moderately higher level of ROS, for example, in cytoplasm, does not destroy the cell membrane—the key feature of necrosis—but mitochondria ROS overproduction may cause inhibition of the cell membrane pumps resulting from reduced ATP synthesis and triggers the necrosis pathway. That interpretation is also consistent with the obtained results and the current knowledge, according to which the target organ in long-term DOX toxicity is the heart but not the liver.

In conclusion, resveratrol had different effects on the oxidative stress and cardiac morphology, which was generally dependent on the dose of doxorubicin. The phytophenol reduced the incidence of cardiac necrosis in rats treated with a higher dose of doxorubicin. It also had an insignificant effect on the hepatic oxidative stress but the substance normalized the organ morphology. In sum, those results give a hope that future studies with higher doses of resveratrol can improve doxorubicin related-toxicity to a broader extent. Due to the number of differences between humans and laboratory rats, the obtained result cannot be directly applied in the human clinical practice. For this reason, more intensive studies, including other nonrodent species, are necessary.

## Figures and Tables

**Table 1:** Serum and plasma markers of heart and liver damage (M ± SD).

	FABP (*μ*g/L)	BNP (*μ*g/L)	AST (IU/L)	ALT (IU/L)	CK (IU/L)	LDH (IU/L)	ALP (IU/L)
Control	5.0 ± 1.59	0.40 ± 0.237	104.3 ± 9.07	57.63 ± 7.11	1536.75 ± 343.61	1275.9 ± 596.29	186.25 ± 31.62
1DOX	7.0 ± 1.97	0.54 ± 0.437	96.0 ± 9.03	68.20 ± 13.65	1099.00 ± 421.51	647.8 ± 179.96	89.80 ± 28.86^a^
1DOX + RV	5.6 ± 0.76	0.52 ± 0.672	130.8 ± 42.49	100.00 ± 23.05^a^	981.80 ± 377.63^a^	979.2 ± 85.56	91.60 ± 19.76^a^
2DOX	11.1 ± 3.26^a^	1.32 ± 0.759^a^	91.4 ± 45.88	48.80 ± 27.69	953.40 ± 142.70^a^	519.2 ± 151.58^a^	84.20 ± 20.09^a^
2DOX + RV	4.6 ± 2.11^b^	0.16 ± 0.257^b^	69.6 ± 18.72^a^	30.00 ± 19.44^a^	545.63 ± 368.32^a^	567.3 ± 211.00^a^	74.63 ± 33.58^a^

^a^ *P* ≤ 0.05 versus control. ^b^ *P* ≤ 0.05 versus DOX.

**Table 2:** Markers of oxidative stress in the heart (M ± SD).

	MDA+4HAE (nmol/g)	GSH/GSSG	GSH_T_ (*μ*mol/g)	SOD (% of control)
Control	18.38 ± 5.16	13.33 ± 5.130	4.30 ± 0.20	100.00 ± 17.525
1DOX	28.46 ± 2.41^a^	11.14 ± 5.142	4.16 ± 0.34	112.22 ± 11.123
1DOX + RV	20.54 ± 2.07^b^	8.83 ± 2.073	4.18 ± 0.19	26.54 ± 10.042^a·b^
2DOX	24.67 ± 2.25^a^	5.65 ± 1.606^a^	4.17 ± 0.26	46.50 ± 19.065^a^
2DOX + RV	33.83 ± 10.40^a^	6.09 ± 1.095^a^	4.14 ± 0.32	65.95 ± 9.606^a^

^a^ *P* < 0.05 versus control, ^b^ *P* < 0.05 versus DOX.

**Table 3:** Markers of oxidative stress in the liver (M ± SD).

	MDA+4HAE (nmol/g)	GSH/GSSG	GSH_T_ (*μ*mol/g)	SOD (% of control)
Control	21.90 ± 5.7	127.36 ± 72.34	13.13 ± 3.34	100.00 ± 34.434
1DOX	31.25 ± 4.91^a^	59.89 ± 83.48	16.05 ± 1.76	92.18 ± 18.579
1DOX+RV	34.54 ± 6.16^a^	162.03 ± 143.16	17.77 ± 3.57	94.47 ± 9.059
2DOX	35.32 ± 9.75^a^	48.59 ± 61.82^a^	10.65 ± 8.22	101.26 ± 27.269
2DOX+RV	33.62 ± 4.24^a^	54.07 ± 53.75^a^	13.21 ± 2.59	70.14 ± 10.647

^a^ *P* < 0.05 versus control.

**Table 4:** Histopathological cardiac changes in animals exposed to doxorubicin (DOX) with or without resveratrol (RV).

	*n*	Eosinophilic degeneration	Parenchymatous degeneration	Vacuolar degeneration	Irregular direction of cardiomyocytes	Pycnotic nuclei of cardiomyocytes	Interstitial edema	Necrosis	Inflammatory infiltration
Control	8	0	1	0	3	0	1	0/0^a^	0/0^b^
1DOX	6	2	3	2	3	1	3	0/4	0/4
1DOX + RV	6	4	4	1	5	0	2	0/2	0/2
2DOX	6	6	4	3	5	2	5	4/2	4/2
2DOX + RV	6	3	2	4	3	2	1	1/3	1/4

A single animal may be represented more than once in the listing of individual histological changes.

^a^Massive necrosis/changes limited to individual cardiomyocytes.

^b^Massive inflammatory infiltration/disseminate mononuclear cells between cardiomyocytes.

**Table 5:** Histopathological hepatic changes in animals exposed to doxorubicin (DOX) with or without resveratrol (RV).

	*n*	Eosinophilic degeneration	Parenchymatous degeneration	Vacuolar degeneration	Cellular edema	Pycnotic nuclei of hepatocytes	Necrosis	Inflammatory infiltration
Control	8	0	2	0	0	0	0/0^a^	0/1^b^
1DOX	6	5	5	0	4	0	0/0	0/0
1DOX + RV	6	4	5	2	0	2	0/0	0/2
2DOX	6	5	6	3	2	3	0/0	0/1
2DOX + RV	6	3	3	2	3	0	0/0	0/2

A single animal may be represented more than once in the listing of individual histological changes.

^a^Massive necrosis/changes limited to single hepatocytes.

^b^Massive inflammatory infiltration/disseminate mononuclear cells between hepatocytes.
